# Efficacy and Safety of Oral Anticoagulants in Patients with Systolic Heart Failure in Sinus Rhythm: A Systematic Review and Meta-analysis of Randomized Controlled Trials and Cohort Studies

**DOI:** 10.1055/s-0040-1720961

**Published:** 2020-11-30

**Authors:** Marie H. Nygaard, Anne-Mette Hvas, Erik L. Grove

**Affiliations:** 1 Department of Cardiology, Aarhus University Hospital, Aarhus, Denmark; 2 Department of Clinical Medicine, Health, Aarhus University, Aarhus, Denmark; 3 Department of Clinical Biochemistry, Thrombosis and Hemostasis Unit, Aarhus University Hospital, Aarhus, Denmark

**Keywords:** heart failure, oral anticoagulants, meta-analysis, sinus rhythm, warfarin, rivaroxaban

## Abstract

**Introduction** There is conflicting evidence on the risk–benefit ratio of oral anticoagulants (OAC) in heart failure (HF) patients without atrial fibrillation. We aimed to evaluate the efficacy and safety of OAC in HF patients in sinus rhythm.

**Methods** A systematic literature search was conducted using PubMed and Embase. We included randomized controlled trials (RCT) and cohort studies, comparing OAC with antiplatelet or no treatment/placebo in patients with HF. Outcomes evaluated were stroke, myocardial infarction (MI), all-cause mortality, and major bleeding.

**Results** Five RCTs and three cohort studies were included. OAC was associated with a reduced risk of ischemic stroke when compared with no treatment/placebo (odds ratio [OR] = 0.67, 95% confidence interval [CI]: [0.47, 0.94]) and antiplatelet therapy (OR = 0.55, 95% CI: [0.37, 0.81]). No significant reduction was found in MI, when OAC was compared with no treatment/placebo (OR = 0.82, 95% CI: [0.63, 1.07]) or antiplatelet therapy (OR = 1.04, 95% CI: [0.60, 1.81]). The all-cause mortality analysis showed no significant reduction when comparing OAC with no treatment/placebo (OR = 0.99, 95% CI: [0.87, 1.12]) or antiplatelet therapy (OR = 1.00, 95% CI: [0.86, 1.16]). The nonsignificant effect of OAC on all-cause mortality was supported by a meta-analysis of the three cohort studies (OR = 1.02, 95% CI: [0.75, 1.38]). Patients treated with OAC had a significantly higher risk of major bleeding than patients receiving antiplatelet therapy (OR = 2.16, 95% CI: [1.55, 3.00]) and a numerically higher risk when compared with no treatment/placebo (OR = 2.38, 95% CI: [0.87, 6.49]).

**Conclusion** The present study does not support the routine use of OAC in patients with HF in sinus rhythm.

## Introduction

Heart failure (HF) affects 26 million people worldwide, and the prevalence is expected to increase. 1 Despite advantages in the management of HF, mortality remains high with a 5-year mortality rate of 40 to 50%. 2 Thus, both frequency and prognosis emphasize the need for improved prophylaxis and treatment of HF and its complications.

HF patients with concomitant atrial fibrillation (AF) are recommended to receive oral anticoagulant (OAC) treatment if there are no contraindications to anticoagulation or increased bleeding risk. 3 Because of the increased thromboembolic risk related to AF, the net clinical benefit of OAC is almost universal among these patients. 3 4 5

Patients with HF in sinus rhythm are predisposed to thromboembolic complications caused by several factors contributing to a hypercoagulable state including the following three components of the Virchow triad: (1) vessel wall abnormalities, (2) abnormal blood constituents, and (3) abnormal blood flow. 6 Increased proinflammatory cytokine levels and neuroendocrine mechanisms also play a significant role in the structural abnormalities in HF. This is manifested by activation of the renin-angiotensinogen-angiotensin pathway and sympathetic nervous systems. 7 This contributes to increase clotting risk and underlines the bidirectional interaction between the coagulation system and inflammatory mechanisms and contributes to the vascular pathogenesis and disease progression seen in HF patients. 8 9

The increased risk of thromboembolic events in patients with HF has led to the assumption that antithrombotic treatment with either OAC or antiplatelet therapy may be beneficial in these patients. 10 11

Importantly, there is conflicting evidence regarding benefits and risks in HF patients without AF. 12 13 Therefore, we investigated the role of OAC treatment in HF patients in sinus rhythm by calculating risk estimates for effect outcomes from published trials. Contrary to previous systematic reviews, we included both randomized clinical trials (RCTs) and cohort studies. We hypothesized that HF patients in sinus rhythm with reduced left ventricular ejection fraction (LVEF ≤ 40%) will benefit from OAC treatment despite the increased risk of major bleeding, when compared with antiplatelet therapy or no treatment/placebo.

## Materials and Methods

### Search Strategy and Study Selection

This systematic review and meta-analysis was conducted in accordance with the Preferred Reporting Items for Systematic Reviews and Meta-analysis (PRISMA) guidelines. 14

PubMed and Embase databases were searched till August 29, 2019. Search filters were English language and human studies. The search strings are listed in the Supplementary Material 1 . M.H.N., E.L.G., and A.M.H. tested the selection strategy by screening 50 random articles by title and abstract based on the preselected characteristics (inclusion and exclusion criteria) to ensure consensus and identify potential disagreements in the search strategy. Disagreements were solved by consensus between the authors. M.H.N. screened and selected all remaining potentially relevant studies, which were further evaluated in full text by all authors.

The PICO strategy consisting of patient, intervention, comparison, and outcome was used as a tool to ensure focused clinical questions. 15 The prespecified criteria for studies included in the meta-analysis were original papers of RCTs or cohort studies of (P) patients over the age of 18 years of either sex with verified systolic HF with reduced LVEF (<40%) in sinus rhythm. Patients should be treated with either (I) OAC monotherapy for a minimum of 3 months with a vitamin-K antagonist (VKA) or a direct oral anticoagulant (DOAC) compared with (C) patients receiving antiplatelet therapy or no treatment/placebo. (O) Outcomes of interest were (i) stroke, (ii) myocardial infarction, (iii) all-cause mortality, and (iv) major bleeding.

In the selection of studies, no restrictions were applied regarding previous treatment, gender, or ethnicity. We excluded reviews, guidelines, editorials, comments without original data, conference abstracts, or case reports with less than 10 cases. Moreover, studies with a follow-up period of less than 6 months or anticoagulant treatment given for less than 3 months were excluded. Studies including patients with HF accompanied by AF were excluded from the meta-analysis, unless these patients only constituted a negligible portion of the study population or presented separately from patients AF.

### Statistical Analysis

Based on data from the individual studies, odds ratios (ORs) with 95% confidence intervals (CIs) were calculated. ORs were pooled for each outcome in a random effects model and statistical heterogeneity assessment between the trials was evaluated using the *I* ^2^ index. Low heterogeneity corresponds to an *I* ^2^ value of <25%, moderate as 50%, and substantial heterogeneity was considered if *I* ^2^ was >75%. 16 Publication bias was visually assessed using the Funnel plot regression. A *p* -value of < 0.05 was considered statistically significant. Meta-analyses were performed, one comparing OAC versus no treatment/placebo and one comparing OAC versus antiplatelet therapy. A separate meta-analysis was conducted for cohort studies on all-cause mortality. A sensitivity analysis was conducted by removing one study (COMMANDER HF 2018). Numbers needed to treat (NNT) or harm (NNH) were calculated on the basis of OR from each meta-analysis and a patient-expected event rate (PEER) estimate, using NNT = (1 − [PEER × (1-or)]) / ([1-peer) × PEER × [1-or]) for ORs greater than 1, and NNT = (1 + [PEER × (or-1)]) / ([1-PEER] × PEER × [or-1]) for OR less than 1. NNH was calculated as ([PEER × [OR-1] + 1) / (PEER × [OR-1] × [1-PEER]). Analyses were performed using Stata Ver. 15 (StataCorp LLC, College Station, Texas).

## Results

### Study Characteristics

**Fig. 1:**
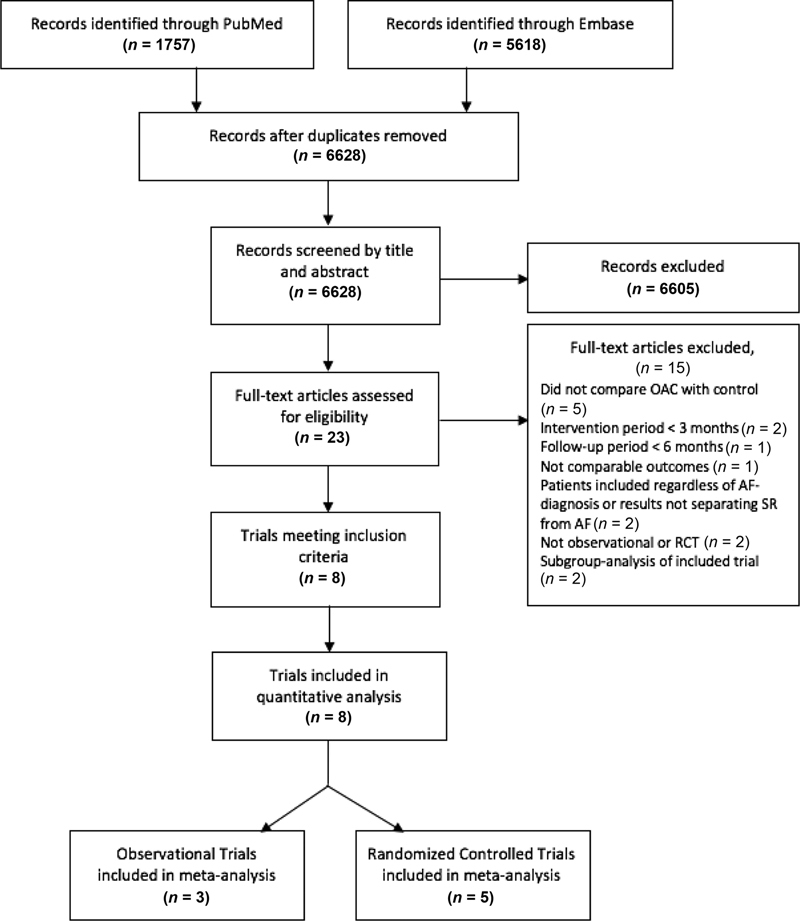
Flow diagram of study selection.

The literature search generated 6,628 records, of which 6,605 were excluded based on title and abstract screening. The remaining 23 records were assessed for eligibility by full-text reading. Eight publications fulfilled the inclusion criteria and were included in the meta-analysis. The process of the study selection is outlined in Fig. 1 .

We included five RCTs and three cohort studies in the analysis with a total of 31,672 eligible participants (9,390 from the RCTs and 22,282 from the cohort trials).

Table 1 summarizes the characteristics of the eight studies included in the meta-analysis. Patient characteristics at baseline are presented in Table 2 .

**Table 1:** Characteristics of included randomized controlled trials and cohort trials investigating the effect of oral anticoagulant treatment in heart failure patients

Study/trial	Design follow up	Patients ( *n* ) type of heart failure	Interventions	Outcomes	Results Odds ratio (OR) and [95% confidence interval]
WASH 2004 (Cleland et al) 17	Multicenter, open-label RCT Mean follow-up: 27 ± 1 months	279 patients LVEF ≤ 35% or end-diastolic internal dimension (≥ 56 mm or ≥30 mm/m ^2^ body surface area) plus a fractional shortening of ≤ 28%	Warfarin (INR 2–3) vs. aspirin (300 mg × 1 day Warfarin vs. no-antithrombotic therapy	Primary: all-cause mortality, nonfatal MI, nonfatal stroke Secondary: death, CV-hospitalization (worsening of HF, MI, stroke, TE-events, major hemorrhage)	Stroke OAC vs. NT: OR = 0.22 [0.01, 4.60], *p* = 0.33 OAC vs antiplatelet: OR= 0.2 [0.01, 4.23], *p* = 0.30 MI OAC vs. NT: OR = 0.46 [0.12, 1.83], *p* = 0.27 OAC vs. antiplatelet: OR = 0.36 [0.09, 1.41], *p* = 0.14 All-cause death OAC vs. NT: OR = 1.22 [0.62, 2.41], *p* = 0.57 OAC vs. antiplatelet: OR = 0.78 [0.40, 1.50], *p* = 0.46 Major bleeding OAC vs. NT: OR = 10.47 [0.56, 197–34], *p* = 0.12 OAC vs. antiplatelet: OR = 4.24 [0.46, 38.66], *p* = 0.20
HELAS 2006 (Cokkinos et al) 18	Multicenter, double-blind RCT, outpatient clinics Mean follow-up: 19.5 months	197 patients LVEF ≤35%, NYHA II–IV, ischemic heart disease	Warfarin (INR 2–3) vs. aspirin (325 mg × 1 day Warfarin vs. placebo	Primary: nonfatal stroke, embolism, MI, rehospitalization, exacerbation of HF, death. Secondary: major bleeding	Stroke OAC vs. placebo: OR = 0.96 [0.08, 10.83], *p* = 0.97 OAC vs. antiplatelet: OR = 0.66 [0.09, 4.78], *p* = 0.68 MI OAC vs. placebo: OR = 2.46 [0.12, 52.31], *p* = 0.56 OAC vs. antiplatelet: OR = 3.40 [0.16, 72.01], *p* = 0.43 All-cause death OAC vs. placebo: OR = 1.04 [0.37, 2.95], *p* = 0.94 OAC vs. antiplatelet: OR = 0.95 [0.38, 2.38], *p* = 0.91 Major bleeding OAC vs. placebo: OR = 7.81 [0.44, 139.86], *p* = 0.16 OAC vs. antiplatelet: OR = 10.79 [0.61, 192.49], *p* = 0.11
WATCH 2009 (Massie et al) 19	Multicenter, RCT, aspirin (double blind), warfarin (open label) Mean follow-up: 1.9 years	1,063 patients a LVEF ≤35%, NYHA II–IV, sinus rhythm	Warfarin (INR 2.5–3.0) vs. aspirin (162 mg × 1 day	Primary: all-cause death, nonfatal MI, nonfatal stroke, major bleeding Secondary: death, nonfatal MI, nonfatal stoke, HF-hospitalization	Stroke OAC vs. antiplatelet: OR = 0.24 [0.07, 0.80], *p* = 0.02 MI OAC vs. antiplatelet: OR = 1.54 [0.86–2.75], *p* = 0.14 All-cause death OAC vs. antiplatelet: OR = 0.94 [0.71, 1.23], *p* = 0.64 Major bleeding OAC vs. antiplatelet: OR = 1.87 [1.11, 3.16], *p* = 0.02
WARCEF 2012 (Homma et al) 20	Multicenter, double-blind RCT Mean follow-up: 3.5 ± 1.8 years	2,305 patients. LVEF ≤35%, NYHA I–IV, planned treatment for HF	Warfarin (INR 2–3.5) Aspirin (325 mg × 1 day	Primary: ischemic stroke, ICH, death Secondary: MI, rehospitalization	Stroke OAC vs. antiplatelet: OR = 0.61 [0.40, 0.94], *p* = 0.03 MI OAC vs. antiplatelet: OR = 0.92 [0.55, 1.54], *p* = 0.75 All-cause death OAC vs. antiplatelet: OR = 1.05 [0.86, 1.27], *p* = 0.63 Major bleeding OAC vs. antiplatelet: OR = 2.24 [1.45, 3.46], *p* < 0.001
COMMAN-DER HF 2018 (Zannad et al) 21	Multicenter, double-blind RCT Mean follow-up: 21.1 months	5,022 patients LVEF ≤40%, >3 months chronic HF, worsening HF within 21 days, CAD, elevated plasma concentrations of natriuretic peptides	Rivaroxaban (2.5 mg × 2 days) vs. placebo	Primary: death, MI, or stroke Secondary: fatal or critical bleeding	Stroke OAC vs. placebo: OR = 0.67 [0.47–0.95], *p* = 0.03 MI OAC vs. placebo: OR = 0.83 [0.63–1.09], *p* = 0.17 All-cause death OAC vs. placebo: OR = 0.98 [0.86, 1.12], *p* = 0.78 Major bleeding OAC vs. placebo: OR = 1.67 [1.17, 2.38], *p* = 0.01
SOLVD 1998 (Al Khadra et al) 22	Retrospective post hoc analysis of RCT Mean follow-up: 3.3 years	6,513 patients. LVEF≤35%	Warfarin vs. no warfarin (no INR target identified)	All-cause death, fatal MI	Stroke OAC vs. NT: OR = 1.35 [0.60, 3.07], *p* = 0.47 MI OAC vs. NT: OR = 0.604 [0.45, 0.80], *p* < 0.001 All-cause death OAC vs. NT: OR = 1.04 [0.88, 1.23], *p* = 0.61
BEST 2011 (Mujib et al) 24	Retrospective post hoc analysis of BEST. Mean follow-up: 2.1 years	1,642 patients. Mean LVEF 23%, all patients in NYHA III/IV	Warfarin vs. no warfarin (no INR target identified)	Primary: all-cause mortality Secondary: cardiovascular and HF mortalities, all-cause and HF hospitalizations	All-cause death OAC vs. NT: OR = 1.35 [1.07, 1.70], *p* = 0.01
ADHERE 2013 (Hernandez et al) 28	Retrospective subgroup analysis of the ADHERE registry linked to Medicare claims Follow-up: 1 year	13,217 patients. LVEF ≤35%, no AF, had not received OAC before admission	Warfarin vs. no warfarin (no INR target identified)	All-cause mortality, thromboembolic events, major adverse cardiovascular events (including major bleeding) and readmission for HF	All-cause death OAC vs. NT: OR = 0.78 [0.68, 0.90], *p* < 0.001 Major bleeding OAC vs. NT: OR = 1.792 [1.35, 2.38], *p* < 0.001

Abbreviations: AF, atrial fibrillation; CAD, coronary artery disease; CV, cardiovascular; HF, heart failure; ICH, intracranial hemorrhage; INR, international normalized ratio; LVEF, left ventricular ejection fraction; MI, myocardial infarction; NT, no treatment; NYHA, New York Heart Association; OAC, oral anticoagulant; RCT, randomized controlled trial; TE, thromboembolic.

a Patients treated with clopidogrel were excluded.

**Table 2:** Baseline characteristics of study participants

	Randomized controlled trials													Cohort studies					
	WASH 2004 17			HELAS 2006 18				WATCH 2009 19		WARCEF 2012 20		COMMANDER HF 2018 21		SOLVD 1998 22		BEST f 2011 24		ADHERE 2013 28	
Intervention	VKA	Asp	NT	IHD e VKA	DCM e VKA	IHD Asp	DCM NT	VKA	Asp	VKA	Asp	Riv	NT	VKA	NT	VKA	NT	VKA	NT
Participants ( *n* )	89	91	99	54	38	61	44	540	523	1,142	1,163	2,507	2,515	861	6,562	471	1,171	1,140	12,077
Male (%)	76	75	72	85	78	93	78	85	85	79.3	80.7	78	76.2	87	85.4	77	76	60.5	53.6
Age (y) Mean ± SD	62	65	61	62.3 ± 9.7	54.8 ± 12.4	61.0 ± 8.9	56.1 ± 11.4	63 ± 11	63 ± 11	61 ± 11.6	61 ± 11.1	66.5 ± 10.1	66.3 ± 10.3	58.5 ± 10.8	59.7 ± 10.1	56 ± 12	60 ± 12	77.0 ± 6.9	78.2 ± 7.6
Mean EF (%) Mean ± SD	NR	NR	NR	28.8 ± 5.9	26.8 ± 5.3	29.3 ± 8.0	27.5 ± 5.5	25 ± 6	24 ± 7	25 ± 7.5	25 ± 7.5	35	34	26.2 ± 6.6	27.1 ± 6.2	21.4 ± 7.3	23.5 ± 7.2	NR	NR
NYHA III–IV (%)	26	29	31	NR	NR	NR	NR	54	58	32.1	29.6	52	53.7	14.7	11.8	91 (III)	93 (III)	NR	NR
AF (%)	7	7	4	0 c	0 c	0 c	0	9.3 d	10.3 d	3.9	3.6	NR	NR	19.3	4.5	0	0	NR	NR
Diabetes (%)	17	19	24	26	11	31	10	38	34	32.6	30.4	40.8	40.9	14.6	20	33	39	35.3	42
Previous MI (%)	NR	NR	NR	84	4	81	0	60	58	48.2	48.7	76.2	75.2	NR	NR	NR	NR	32.2	38.7
Hypertension (%)	34	30	37	45	24	48	25	49	48	60.8	61.7	75.7	75	34.4	39.8	51	61	67.7	71.2
Systolic BP (mm Hg) Mean ± SD	126	124	127	NR	NR	NR	NR	120 ± 19	118 ± 18	124 ± 19.3	124 ± 18.4	NR	NR	NR	NR	114 ± 16	119 ± 19	NR	NR
Nonsmoker (%)	NR	NR	NR	48	63	38	60	21	21	30.2	31.4	NR	NR	21.8	21.7	80	82	NR	NR
Use of ACE-I/ARB (%)	90 a	88 a	94 a	51.2	65.3	62	66.5	88/11	88/11	98.4	98.4	93.6	92.0	NR	NR	97	97	76.4	72.1
Use of diuretics (%)	98 b	96 b	93 b	52	61	60	64	98	98	81.4	80.3	99.5	99.6	48.8	41.8	92	93	80.9	78.1
Use of β-blocker (%)	10	8	14	12.5	5.5	17.4	8.9	71	69	90.3	98.5	91.7	93.1	13.3	18.6	49	51	71.9	68.4
Baseline aspirin/APT (%)	56	42	46	NR	NR	NR	NR	NR	NR	7.5	8.7	92.9	93.3	17.7	50.9	21	63	46.2	64.4
Baseline warfarin (%)	4	9	2	NR	NR	NR	NR	1	1	7.9	7.7	NR	NR	NR	NR	NR	NR	NR	NR

Abbreviations: ACE-I, angiotensin-converting enzyme inhibitor; AF, atrial fibrillation; APT, antiplatelet therapy; ARB, angiotensin II receptor blocker; asp, aspirin; BP, blood pressure; DCM, dilated cardiomyopathy group; EF, ejection fraction; IHD, ischemic cardiomyopathy group; MI, myocardial infarction; N, number; NR, not reported; NT, no treatment; NYHA, New York Health Association; SD, standard deviation; VKA, vitamin-K antagonist.

a ACE only.

b Loop diuretics.

c AF was an absolute contraindication. Three developed AF but was excluded.

d During follow-up.

e IHD/warfarin and DCM/warfarin groups were combined to one group in the meta-analysis.

f Before propensity making.

Of the 9,390 patients in the RCTs, 4,370 were allocated to OACs (1,863 to VKA and 2,507 to DOAC), 99 to no treatment, 2,559 to placebo, and 1,838 to antiplatelet therapy. The mean follow-up time averaged from 1.9 to 3.5 years in the RCTs. The reported mean LVEF was 27.7%, the mean age of the participants across the RCTs were 61.8 years, and the majority (72–93%) population were men.

The five RCTs included in the meta-analysis were WASH (the warfarin/aspirin study in HF) 2004, 17 HELAS (HF long-term antithrombotic study) 2006, 18 WATCH (warfarin and antiplatelet therapy in HF) 2009, 19 WARCEF (warfarin and antiplatelet therapy in chronic HF) 2012, 20 COMMANDER HF (a study to assess the effectiveness and safety of rivaroxaban in reducing the risk of death, myocardial infarction, or stroke in participants with HF and coronary artery disease following an episode of decompensated HF) 2018. 21 The three cohort studies included in analysis were; SOLVD (studies of left ventricular dysfunction) 1998, 22 BEST (beta-blocker evaluation of survival trial) 2011, 23 and ADHERE (acute decompensated heart failure national registry) 2013. 24 Of the total 22,282 patients in the cohort studies, 2,472 were patients receiving OAC, whereas 19,810 patients received no OAC.

### Meta-analysis Results

**Fig. 2:**
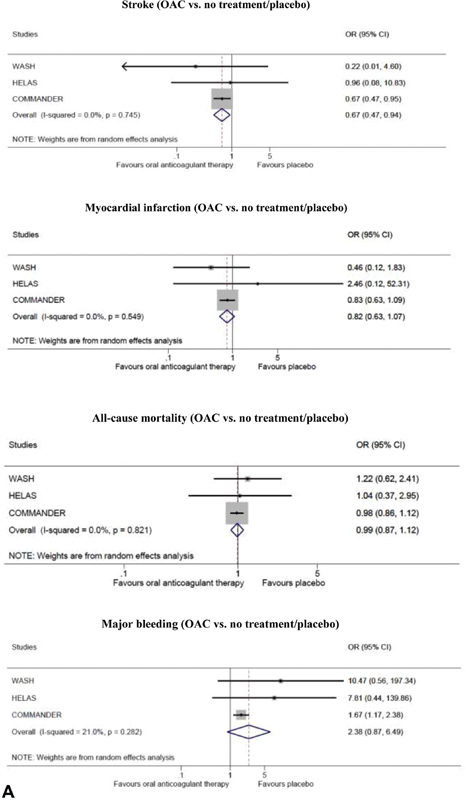
( **A** ) Outcomes of oral anticoagulant (OAC) treatment versus no treatment/placebo in randomized controlled trials; stroke, myocardial infarction, all-cause mortality and major bleeding. ( **B** ) Outcomes of oral anticoagulant treatment versus antiplatelet therapy in randomized controlled trials; stroke, myocardial infarction, all-cause mortality and major bleeding. ( **C** ) All-cause mortality of oral anticoagulant treatment versus no treatment in cohort studies. ( **D** ) Sensitivity analysis for outcomes of oral anticoagulant treatment versus no treatment/placebo in randomized controlled trials without the COMMANDER HF 2018 study; stroke, myocardial infarction, all-cause mortality and major bleeding. CI, confidence interval; COMMANDER HF, a study to assess the effectiveness and safety of rivaroxaban in reducing the risk of death, myocardial infarction, or stroke in participants with HF and coronary artery disease following an episode of decompensated heart failure; OR, odds ratio.

The five RCTs contributed to the meta-analysis of the four outcomes of interest; stroke, MI, all-cause mortality, major bleeding, and the safety outcome. Fig. 2A shows the comparison of OAC with no treatment/placebo and Fig. 2B depicts OAC compared with antiplatelet therapy.

**Figure:**
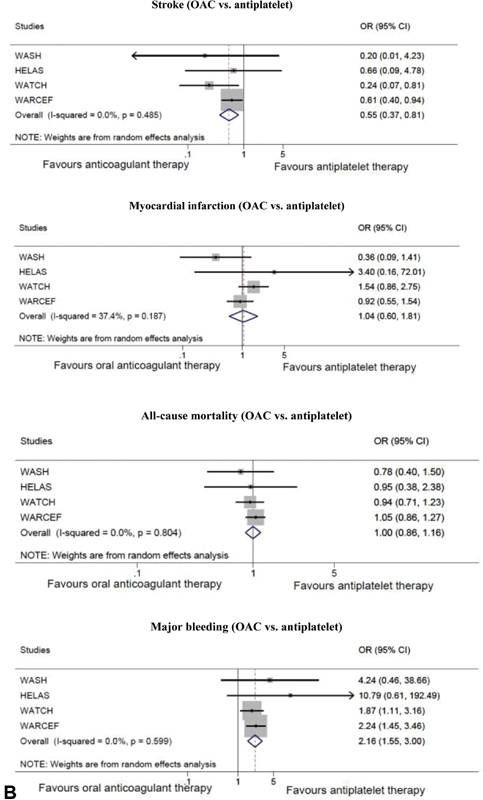


**Figure:**
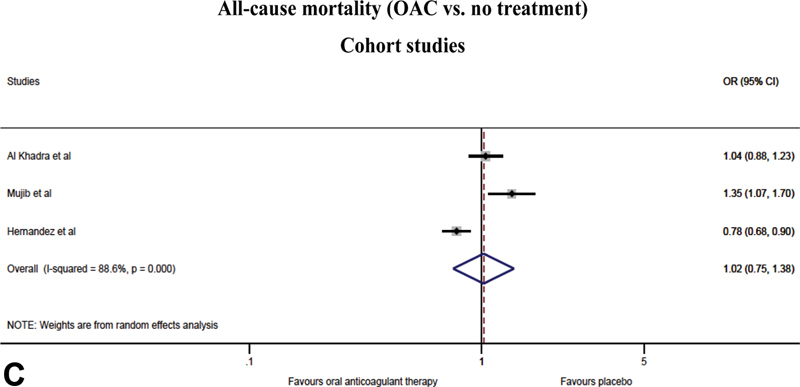


**Figure:**
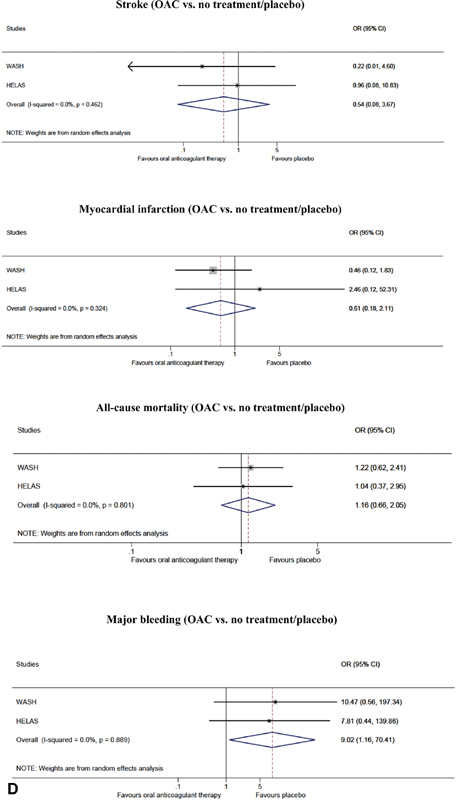


Stroke was not equally reported in the trials, with all stroke data reported in the WATCH 2009, nonfatal stroke in HELAS 2006, and WASH 2004, and both ischemic and hemorrhagic stroke reported in WARCEF 2012. 17 18 19 20 MI was unspecified in the WARCEF 2012 and COMMANDER HF 2018 trials, nonfatal MI was reported in WATCH 2009 and HELAS 2006, and both fatal and nonfatal MI in the WASH 2004 trial. 17 18 19 20 21 All-cause mortality was reported in all five RCTs. 17 18 19 20 21 Major bleeding was defined as requirement of blood transfusion or bleeding leading to disability or death.

All three cohort studies contributed to the outcome of all-cause mortality. 22 23 24 The data on stroke and MI was not specifically provided in the BEST 2011 and ADHERE 2013 trials, and only the ADHERE 2013 study reported major bleeding events. 23 24 Fig. 2C shows the meta-analysis of all-cause mortality in the cohort studies.

In the COMMANDER HF 2018 study, treatment with OAC differed from traditional OAC with respect to the anticoagulant agent (rivaroxaban) and dosage (2.5-mg twice daily which is lower than the usual 15 or 20 mg of daily rivaroxaban). Therefore, we performed an additional sensitivity analysis for OAC versus no treatment/placebo without the COMMANDER HF 2018 study ( Fig. 2D ).

Funnel plots were assessed for each of the outcomes evaluated ( Supplementary Material 2 ).

#### Stroke

OAC was associated with a significant reduction in stroke when compared with no treatment/placebo (OR = 0.67, 95% CI: [0.47, 0.94]; NNT = 104) and antiplatelet therapy (OR = 0.55, 95% CI: [0.37, 0.81]; NNT = 63). The *I* ^2^ test revealed no heterogeneity in stroke outcomes between the studies ( *I* ^2^ = 0.0% and *p* < 0.05 in both analyses). Funnel plot for stroke did not show any risk of significant publication bias. The sensitivity analysis excluding the COMMANDER HF 2018 study showed a lower risk of stroke in patients treated with OAC compared with no treatment/placebo, although this was not significant (OR = 0.54, 95% CI: [0.08, 3.67]).

#### Myocardial Infarction

There was no significant reduction in MI when OAC was compared with either no treatment/placebo (OR = 0.82, 95% CI: [0.63, 1.07]; NNT = 123) or antiplatelet therapy (OR = 1.04, 95% CI: [0.60, 1.81]; NNT = 925). This was a consistent finding in all five RCTs. Moderate heterogeneity was acknowledged in the OAC versus antiplatelet therapy analysis but was not significant ( *I* ^2^ = 37.4% and *p* = 0.187). No evidence of significant bias was found in the funnel plot for MI. The sensitivity analysis excluding the COMMANDER HF 2018 study did not show any changes in the outcome of MI when OAC therapy was compared with no treatment/placebo (OR = 0.61, 95% CI: [0.18, 2.11]).

#### All-Cause Mortality

With respect to all-cause mortality, the meta-analysis of the RCTs showed no effect of OAC compared with antiplatelet therapy (OR = 1.00, 95% CI: [0.86, 1.16]; NNT not possible to calculate due to OR = 1.00) or no treatment/placebo (OR = 0.99, 95% CI: [0.87, 1.12]; NNT = 583). A similar lack of effect was found in the meta-analysis of the cohort studies (OR = 1.02, 95% CI: [0.75, 1.38]; NNT = 243). No significant heterogeneity was found in the analysis of the RCTs ( *I* ^2^ = 0.0%, *p* = 0.821 for OAC vs no treatment and *I* ^2^ = 0.0%, *p* = 0.804 for OAC vs. antiplatelet therapy) whereas significantly high heterogeneity was found between the cohort studies ( *I* ^2^ = 88.6%, *p* < 0.00). We found no indication of bias with the outcome all-cause mortality (RCTs only), whereas the funnel plot for all-cause mortality indicated the possibility of some bias. However, with only three studies in the analysis, this should be interpreted with caution. The sensitivity analysis excluding the COMMANDER HF 2018 study did not show any changes in the outcome of all-cause mortality when OAC was compared with no treatment/placebo (OR = 1.16, 95% CI: [0.66, 2.05])

#### Major Bleeding

When analyzing the risk of major bleeding between groups of the RCTs, there was a doubled risk of major bleeding in the OAC group compared with the antiplatelet group (OR = 2.16, 95% CI: [1.55, 3.00]; NNH = 35) and to no treatment/placebo (OR = 2.38, 95% CI: [0.87, 6.49]; NNH = 40), although the latter was not significant. No heterogeneity was found in the OAC vs antiplatelet therapy ( *I* ^2^ =0.0%, *p* = 0.599), and low heterogeneity was found between the studies in the OAC versus no treatment analysis ( *I* ^2^ = 21.0%, *p* = 0.282). The funnel plot for major bleeding showed that the smaller studies were located in the bottom right quadrant, thus indicating the possibility of some (publication) bias. The sensitivity analysis excluding the COMMANDER HF 2018 study showed a nine-fold increased risk of major bleeding in the OAC group compared with the no treatment/placebo group (OR = 9.02, 95% CI: [1.16, 70.41]).

## Discussion

The present meta-analysis of five RCTs comprising 9,390 participants showed that patients with HF in sinus rhythm receiving OAC therapy had a lower risk of stroke than patients receiving antiplatelet therapy or no treatment/placebo. Conversely, an increased risk of major bleeding was seen based on the calculated ORs of all five RCTs. No benefit was found from OAC as regard to all-cause mortality and MI. The lack of benefit from OAC on all-cause mortality was supported by an additional meta-analysis of three cohort studies with a total of 22,282 patients.

The role of OAC in HF patients in sinus rhythm has been a subject of discussion for many years with conflicting conclusions in previous studies. The first trials performed more than 60 years ago reported favorable effects of OAC in HF with reduced rates of embolic events and deaths. 25 26 27 However, these results should be interpreted with caution, because many of the included patients suffered from comorbid conditions as AF and valvular heart disease, which increases the risk of thromboembolic events. Previous reviews of the literature provide no clear evidence that treatment with OAC is beneficial for HF patients in sinus rhythm. 28 29 30

The present systematic review revealed minor heterogeneity between the conducted RCTs; Three studies were double blinded, whereas two were open label. The study populations did not differ markedly with respect to age, gender, follow-up time, and systolic blood pressure. On the other hand, the size of the study populations differed substantially from 279 in WASH to 5,022 in COMMANDER HF, making the results primarily driven by the COMMANDER HF trial. 17 21 COMMANDER HF differs from the other trials both regarding the anticoagulant agent and dosage, as the COMMANDER HF used low-dose rivaroxaban 2.5-mg twice daily, 21 whereas the remaining trials used “full-dose” warfarin with international normalized ratio (INR) targets provided in Table 1 . These differences in use of drugs and dosages across the studies represent possible confounding effects on the meta-analysis. The sensitivity analysis conducted after removing the COMMANDER HF strengthens the conclusion that patients with HF in sinus rhythm does not benefit from OAC. Accordingly, no overall benefit was found from OAC as regard to the outcomes stroke, all-cause mortality and MI. On the other hand, there was a significantly increased risk of major bleeding among patients receiving OAC.

The mean reported LVEF varied between 24 and 35% across the RCTs. 17 18 19 20 21 Low LVEF in HF patients is an independent risk factor itself, and an inverse relationship between the risk of stroke and LVEF has been shown. 31 The SAVE (Survival and Ventricular Enlargement trial) study showed an 18% increase in stroke risk per 5% LVEF reduction and two-fold higher risk when EF was below 28% compared compared with patients with EF > 28%. 32 However, a population-based 30-year cohort study reported a higher risk of all subtypes of stroke among HF patients than in the general population, with a 1- and 5-year risk of ischemic stroke of 1.4 and 3.9%, respectively. 11 The INR target in the WASH and HELAS trials was 2.0 to 3.0 with a mean INR in the WASH study of 2.3, but none of the studies reported the time in therapeutic range. 17 18 A subgroup analysis of the WARCEF trial showed that increasing time in therapeutic range was associated with improved net clinical benefits. 33 Time in therapeutic range was not reported in the cohort studies because of their retrospective data collection. 22 23 24 Thus, an underestimation of the effect of OAC cannot be excluded.

Primarily because of poor recruitment and dropouts, the RCTs were not able to attain the prespecified enrolment numbers and were stopped early, 17 18 19 20 21 thus making them underpowered to make conclusive statements. The HELAS trial excluded patients with concomitant AF, but 10% of the patients in the WATCH trial developed AF during follow-up. 18 19 In the WASH and WARCEF trials 6.4 and 3.7% participants, respectively, had AF at baseline with the WASH study reporting separate outcomes of patients without AF. 17 20 The occurrence of subclinical AF is possible in all RCTs, because of no electrocardiographic monitoring during follow-up. 17 18 19 20 21 AF was only present in 0.9% of all participants in this RCT meta-analysis. Thus, we do not expect the overall conclusion to be affected by this.

In the cohort studies, the prevalence of AF differed between studies. In the SOLVD study, 19.3% in the warfarin arm versus 4.5% in the non–warfarin arm suffered from AF. 22 In the BEST study, AF was an exclusion criterion, whereas the proportion of AF was not reported in the ADHERE study. 23 24 In the SOLVD study, many patients had a relatively low New York Heart Association (NYHA) class with only 12% of patients having symptom severity comparable to NYHA classes III and IV. 22 34 In the BEST 2011 study, all patients were in NYHA III/IV with a mean EF of 23%, and thereby had more severe HF. 23 The proportion of patients taking antiplatelet therapy at baseline was generally high, primarily in the warfarin arms, and differed across trials with 47% in the SOLVD study and 62.8% in the ADHERE study. 22 23

A meta-analysis will always be affected by the limitations of the individual studies. Substantial heterogeneity exists between the cohort studies included in the analysis of all-cause mortality, which is to be expected based on their designs. Although the heterogeneity between the RCTs was low to moderate (37% in MI, 89% in all-cause mortality, and 21% in major bleeding), significant heterogeneity was absent in the analysis of the RCTs which support the conclusions from our meta-analysis.

The most important limitations of the RCTs are the premature termination of all five RCTs, suboptimal blinding, and the use of different OAC drugs and dosages across the studies. Another shortcoming is the lack of individual patient data that precluded exploration of the efficacy and safety of OACs in different subgroups. The inclusion of both RCTs and cohort studies increased the number of study participants, although the cohort studies only contributed to an analysis of all-cause mortality. The cohort trials have several limitations, including lack of randomization, unknown reason for OAC prescription, and no report of the quality of anticoagulant therapy. Additionally, given the fact that we chose to include studies with HF patients with reduced LVEF, the results cannot be extrapolated to patients with HF and preserved or midrange LVEF.

Current guidelines recommend no routine use of OAC in HF patients in sinus rhythm, whereas patients with evidence of AF and/or underlying conditions predisposing to venous thromboembolism should be considered for OAC treatment. 5 There is considerable heterogeneity among HF patients, and it is conceivable that specific HF subgroups will derive more benefit from OAC. It is possible that the study population in our meta-analysis is heterogenous with respect to thromboembolic risk, thus masking a potential net clinical benefit of OAC in higher risk patients. Future clinical trials are needed to identify high-risk subgroups who may benefit from OAC.

The CHA _2_ DS _2_ -VASc and HAS-BLED scores have been developed as useful tools to stratify the risk of stroke and bleeding, and these scoring systems are recommended in AF guidelines. In recent years, the CHA _2_ DS _2_ -VASc score has been proposed to predict cardiovascular outcomes in other non-AF clinical settings. From this perspective, a high score could potentially justify OAC treatment in patients even without AF, but further research is needed to establish a similar risk stratification scheme for HF patients with sinus rhythm. A risk score may predict the risk of stroke and bleeding risk and guide the need for OAC in HF patients with sinus rhythm. 35 Additionally, the effect of low-dose rivaroxaban and other DOACs on HF patients in sinus rhythm needs to be further explored.

## Conclusion

The present study demonstrates a reduced risk of ischemic stroke among HF patients in sinus rhythm treated with OAC, but treatment with OAC also increases the risk of major bleeding. Thus, based on the results of the present meta-analysis supported by a sensitivity analysis, routine OAC treatment is not justified in patients with HF in sinus rhythm, unless indicated for other cardiovascular conditions. Future studies may be able to identify HF patients with an increased risk of stroke who based on, for example, risk scores or echocardiography findings are likely to benefit from anticoagulant treatment.
