# Supplementary material for: Efficacy and Safety of Oral Anticoagulants in Patients with Systolic Heart Failure in Sinus Rhythm: A Systematic Review and Meta-analysis of Randomized Controlled Trials and Cohort Studies
Source: TH Open. 2020 Nov 30;4(4):e383–92. doi: 10.1055/s-0040-1720961 (PMC7704246; doi:10.1055/s-0040-1720961)

Supplementary Material 1: Search Strings

Database	Search string
Embase	"heart failure" AND ("anticoagulation" OR "rivaroxaban" OR "dabigatran" OR "apixaban" OR "edoxaban" OR "warfarin") AND [article]/lim AND [humans]/lim AND [English]/lim
PubMed	("heart failure" OR "Heart Failure"[Mesh]) AND (anticoagula* OR "Anticoagulants"[Mesh] OR rivaroxaban OR apixaban OR edoxaban OR dabigatran OR warfarin) NOT "Atrial Fibrillation"[Mesh] NOT "Review" [Publication Type] NOT "Guideline" [Publication Type] NOT "Editorial" [Publication Type] NOT "Comment" [Publication Type]) NOT "Congress" [Publication Type].

Supplementary Material 2: Funnel Plots

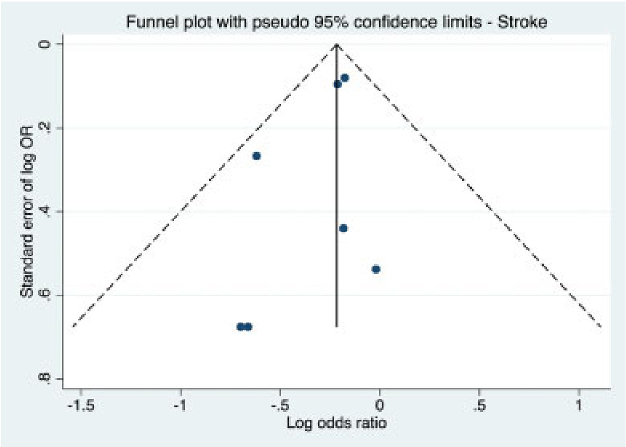

Funnel plots (RCTs only). MI, myocardial infarction; OR, odds ratio; RCT, randomized controlled trial.

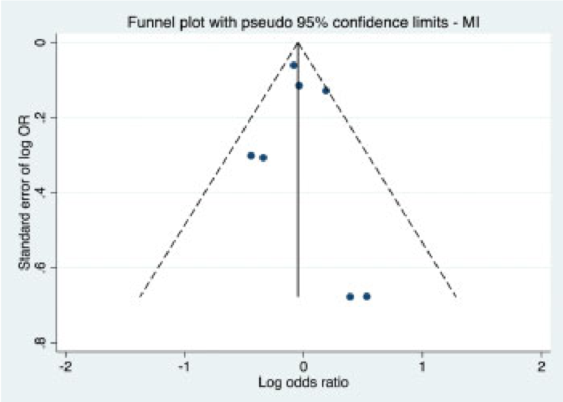

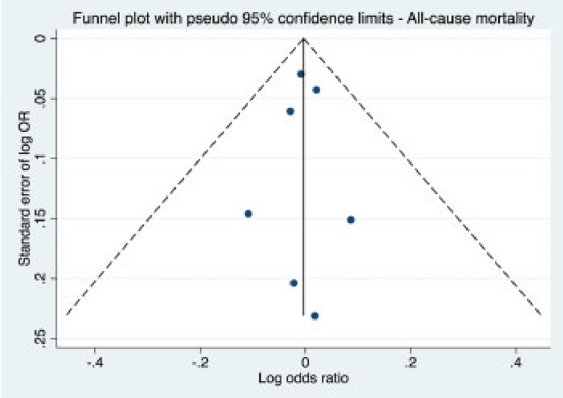

Funnel plots (cohort studies only). OR, odds ratio.

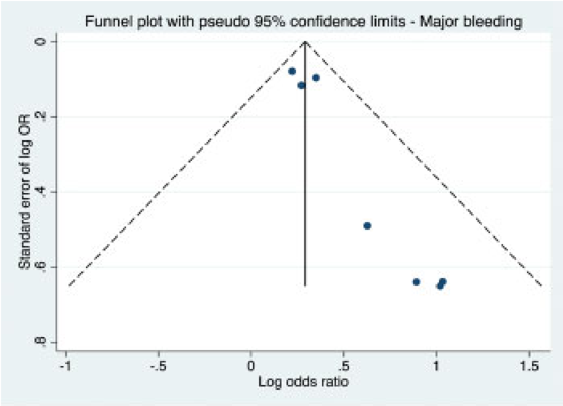

Funnel plots (cohort studies only). OR, odds ratio.

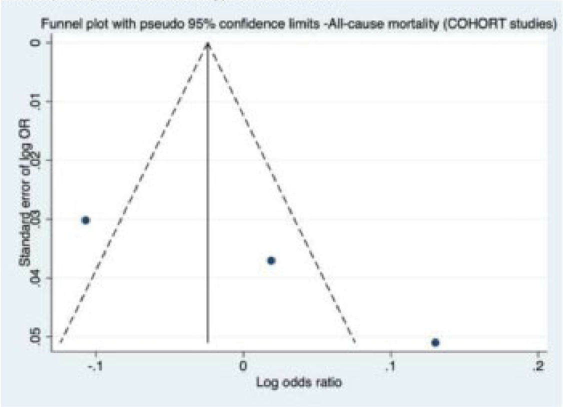

Supplement: Supplementary file 1 — Supplementary Material [file 10-1055-s-0040-1720961-s200030.pdf]
